# The effects of conjugate and light dose on photo-immunotherapy induced cytotoxicity

**DOI:** 10.1186/1471-2407-14-389

**Published:** 2014-05-30

**Authors:** Takahito Nakajima, Kazuhide Sato, Hirofumi Hanaoka, Rira Watanabe, Toshiko Harada, Peter L Choyke, Hisataka Kobayashi

**Affiliations:** 1 Molecular Imaging Program, Center for Cancer Research, National Cancer Institute, NIH, Bethesda, Bldg. 10, Room B3B69, MSC 1088, Bethesda, Maryland 20892-1088, USA

**Keywords:** Photoimmunotherapy, Near infrared light, Light dose, Necrosis, Cytotoxicity

## Abstract

**Background:**

Photoimmunotherapy (PIT) is a highly cell-selective cancer therapy, which employs monoclonal antibodies conjugated to a potent photosensitizer (mAb-IR700). Once the conjugate has bound to the target cell, exposure to near infrared (NIR) light induces necrosis only in targeted cells with minimal damage to adjacent normal cells *in vivo*. Herein, we report on the effect of altering mAb-IR700 and light power and dose on effectiveness of PIT.

**Methods:**

For evaluating cytotoxicity, we employed ATP-dependent bioluminescence imaging using a luciferase-transfected MDA-MB-468luc cell line, which expresses EGFR and luciferase. In *in vitro* experiments, panitumumab-IR700 (Pan-IR700) concentration was varied in combination with varying NIR light doses administered by an LED at one of three power settings, 100 mA and 400 mA continuous wave and 1733 mA intermittent wave. For *in vivo* experiments, the MDA-MB-468luc orthotopic breast cancer was treated with varying doses of Pan-IR700 and light.

**Results:**

The *in vitro* cell study demonstrated that PIT induced cytotoxicity depended on light dose, when the conjugate concentration was kept constant. Increasing the dose of Pan-IR700 allowed lowering of the light dose to achieve equal effects thus indicating that for a given level of efficacy, the conjugate concentration multiplied by the light dose was a constant. A similar relationship between conjugate and light dose was observed *in vivo*.

**Conclusions:**

The efficacy of PIT is defined by the product of the number of bound antibody conjugates and the dose of NIR light and can be achieve equally with continuous and pulse wave LED light using different power densities.

## Background

Photoimmunotherapy (PIT) is a highly cell-selective cancer therapy, which utilizes a monoclonal antibody (mAb) conjugated to the photosensitizing phthalocyanine dye, IRDye700DX (IR700). After intravenous injection, mAb-IR700 conjugates preferentially to cancer cells expressing the proper antigen and subsequent exposure of the cells to near infrared (NIR) light induces highly selective and rapid cell necrosis. An attractive feature of PIT is that minimal damage is seen in adjacent normal cells [1]. The effectiveness of PIT appears generalizable across a number of different types of cancers and with multiple mAb-IR700 conjugates [2]. When the mAb-IR700 conjugate is well matched to the target tumor, exposure to NIR light results in rapid and severe damage to the cell membrane inducing necrotic cell death within a minute [3]. However, the relationship between the dose of mAb-IR700 and the dose or power density of NIR light has not been fully investigated. Optimized dosing of NIR light exposure is critical for planning a successful clinical trial of PIT in both therapeutic efficacy and patient safety.

PIT induces cell death by necrosis that releases ATP from cells, which is quickly hydrolyzed. Thus, we employed ATP-dependent bioluminescence imaging of firefly luciferase transfected cells as a readout of the effectiveness of PIT while varying the dose of the mAb-IR700 and NIR light that was proven to be a more accurate biomarker for accessing acute PIT effects than tumor size especially within 3 days after PIT [4,5].

## Methods

### Reagents

Panitumumab, a fully humanized IgG2 monoclonal antibody (mAb) directed against human EGFR was purchased from AMGEN Inc. (Thousand Oaks, CA). A water soluble,silicon-based-phthalocyanine dye derivative, IRDye 700DX NHS ester (IR700; C_74_H_96_N_12_Na_4_O_27_S_6_Si_3_, molecular weight of 1954.22), was obtained from LI-COR Bioscience (Lincoln, NE). All other chemicals used were of reagent grade.

### Synthesis of IR700-conjugated Panitumumab

Panitumumab (1 mg, 6.8 nmol) was incubated with IR700 (66.8 μg, 34.2 nmol, 5 mmol/L in DMSO) in 0.1 mol/L Na_2_HPO_4_ (pH 8.5) at room temperature for 1 h. Then the mixture was purified with a Sephadex G50 column (PD-10; GE Healthcare, Piscataway, NJ). The protein concentration was determined with a Coomassie Plus protein assay kit (Pierce Biotechnology, Rockford, IL) by measuring the absorption at 595 nm (8453 Value System; Agilent Technologies, Santa Clara, CA). The concentration of IR700 was measured by its absorption to confirm the number of fluorophore molecules conjugated to each Panitumumab molecule. The number of IR700 per antibody was approximately 4 for the 1:4.5 reaction conditions. The resulting compound, Pan-IR700, was kept at 4°C as a stock solution.

### Cell line

EGFR-expressing MDA-MB-468luc, [6] stable luciferase-transfected cells were grown in RPMI 1640 supplemented with 10% fetal bovine serum and 1% penicillin/streptomycin in tissue culture flasks using a humidified incubator at 37°C in an atmosphere of 95% air and 5% carbon dioxide.

### Fluorescence microscopy

To detect the antigen specific localization of Pan-IR700, fluorescence microscopy was performed (BX61; Olympus America, Melville, NY). MDA-MB-468luc was seeded on cover-glass-bottomed dishes and incubated for 16 h. Pan-IR700 conjugate (10 μg/mL) was added to the culture medium and incubated for 6 h at 37°C, followed by washing with PBS. The filter was set to detect IR700 fluorescence with a 590–650 nm excitation filter and a 665–740 nm band pass emission filter.

### Phototoxicity assay

Cytotoxicity of PIT was determined by measuring luciferase activity and by quantitative flow cytometry using propidium iodide (PI) as a stain for dead cells. For the luciferase activity assay, d-luciferin (150 μg/ml, Gold Biotechnology, St. Louis, MO) was added to the culture medium and bioluminescence (signal intensity) was detected (Photon Imager; Biospace Lab, Cambridge, MA). For the flow cytometry assay, cells were trypsinized after treatment, washed with PBS and 1 μL of PI (5 mg/mL) was added to the cell suspension which was vortexed. Cells were analyzed on a flow cytometer (FACS Calibur; BD BioSciences, San Jose, CA).

### *In vitro* photoimmunotherapy

**Figure 1:**
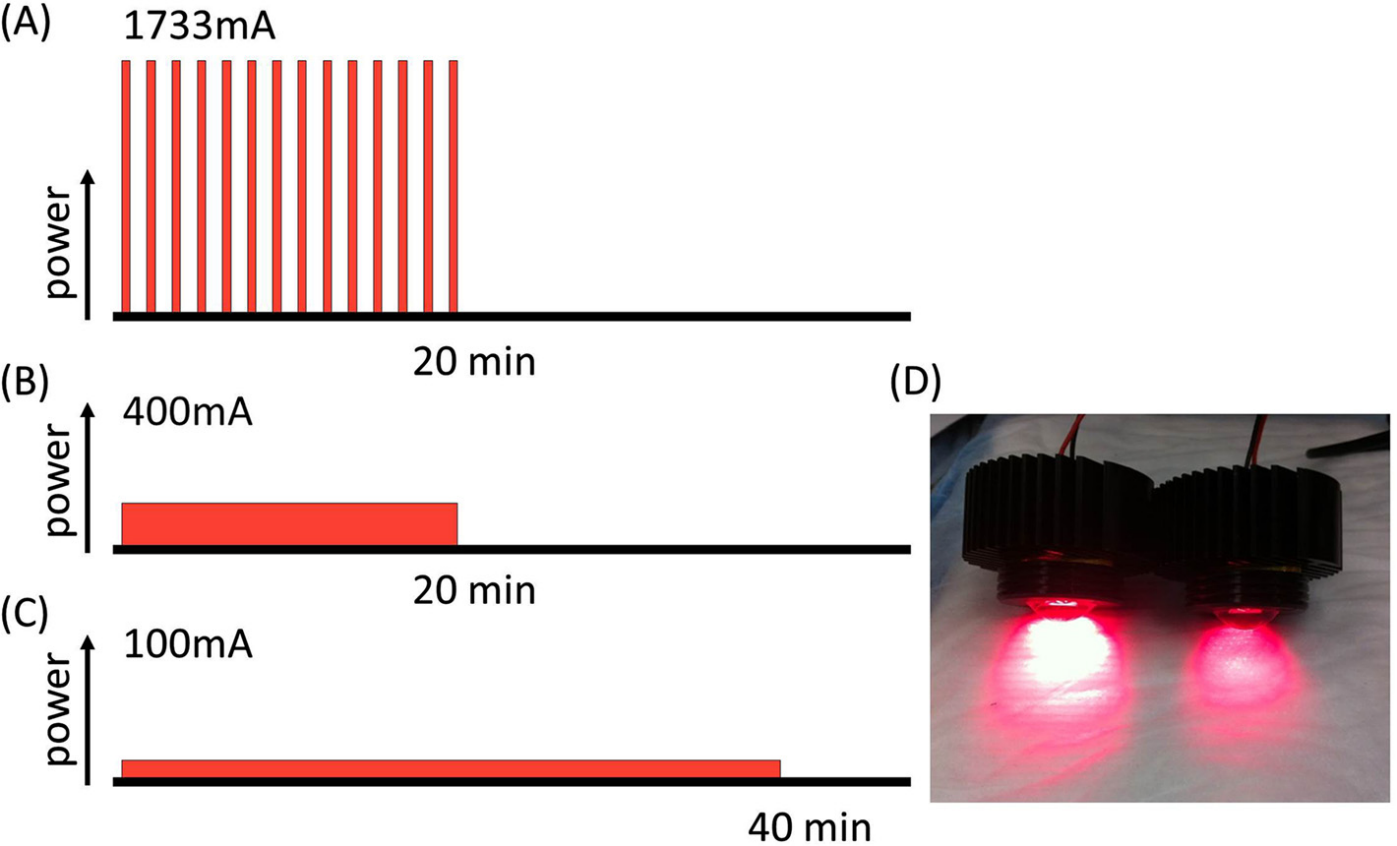
**Schematic sequences of LED irradiation. (A)** Pulse wave (PW) lighting is achieved via peak currents of 1733 mA. **(B)** Continuous wave (CW) lighting at 400 mA and 100 mA. **(C)** The irradiation times were 20 min for the 1733 mA (PW) and 400 mA (CW) and it took 40 min for irradiation at 100 mA (CW). Total irradiation dose of these three groups was adjusted to 30 J/cm^2^. **(D)** Photograph of LED lights, left; 1733 mA (PW), right; 400 mA (CW).

Cells were seeded on a 96 well plate or 35 mm cell culture dishes and incubated for 8 h at 37°C. The culture medium was refreshed and 10 μg/mL of Pan-IR700 was added over night. After washing with PBS, phenol red free culture medium was added. Then, cells were irradiated with a red light-emitting diode (LED), which emits light at 670 to 710 nm wavelength (L690-66-60; Marubeni America Co., New York, NY), controlled by FluorVivo software (INDEC Systems, Santa Clara, CA) at a current of 100 mA (continuous-wave; CW), 400 mA (CW) and 1733 mA (pulse-wave; PW). The pulse wave duration was 0.2 ms separated by 0.8 ms so that the pulse occurred every 1 ms (Figure 1). The power density of the LED was 12.5 mW/cm^2^ at 100 mA CW and 25 mW/cm^2^ at 400 mA CW and 1733 mA PW as measured with an optical power meter (PM 100; Thorlabs, Newton, NJ).

MDA-MB-468luc cells were irradiated at 0.2, 0.5, 2 and 5 J/cm^2^ using all 3 power settings (100 mA CW, 400 mA CW and 1733 mA PW). Cell viability was analyzed with flow cytometry and bioluminescence imaging.

Pan-IR700 was added to cells at concentrations of 0.3, 1, 3, 10 μg/mL. Cells were incubated for 8 h followed by washing once with PBS and restoration of phenol red free culture medium. The cells were then irradiated with the LED light of 400 mA (CW) at a total dose of 2 J/cm^2^. *In vitro* treatments for cells were performed using the following combinations of Pan-IR700 and NIR light dose: (1) 3 μg/mL and 0.5 J/cm^2^, (2) 1 μg/mL and 1.5 J/cm^2^, and (3) 0.3 μg/mL and 5 J/cm^2^.

### Tumor model

All procedures were carried out in compliance with the Guide for the Care and Use of Laboratory Animal Resources (1996), National Research Council, and approved by the local Animal Care and Use Committee. Six- to eight-week-old female homozygote athymic nude mice were purchased from Charles River (NCI-Frederick, Frederick, MD). During the procedure, mice were anesthetized with isoflurane. MDA-MB-468luc cells (2 × 10^6^ cells) were injected subcutaneously into the right mammary pads of the mice. The experiments were conducted 2 weeks after MDA-MB-468luc cell implantation.

### *In vivo* photoimmunotherapy with different power levels of LED light

Orthotopic breast tumors were irradiated at all three power settings, 100 mA (CW), 400 mA (CW) and 1733 mA (PW). Total irradiation doses were 30 J/cm^2^ with power density of 200 mW/cm^2^. Mice images were acquired with a fluorescence imager (Pearl Imager; LI-COR Biosciences) for detecting IR700 fluorescence, and Photon Imager for BLI. BLI was used for evaluation of PIT effects. Regions of interest (ROIs) were placed over the entire tumor and photon numbers were counted for each ROI.

### Statistical analysis

Statistical analysis was performed using a statistics program (GraphPad Prism6, GraphPad Software, La Jolla, CA). A one-way analysis of variance (ANOVA) was used to compare differences in responses to level of light exposure among the three groups. Pearson’s correlation coefficient was used to analyze the correlation between the dead cell ratio and the concentrations of Pan-IR700. Values of p < 0.05 were considered statistically significant.

## Results

### Target specific binding of Pan-IR700 to EGFR on fluorescence microscopy

**Figure 2:**
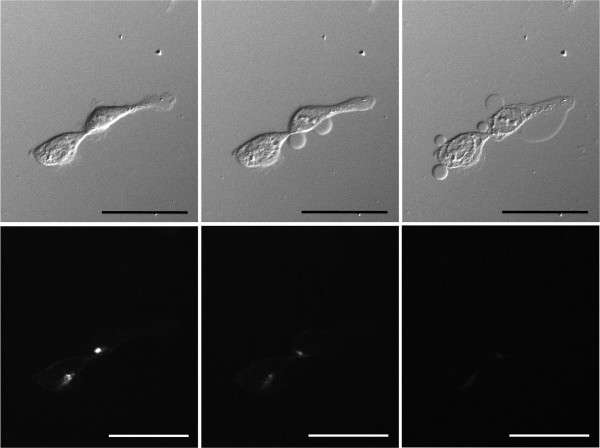
**Sequential microscopic images of MDA-MB-468luc cells treated with Pan- IR700 (images before (left), during (middle) and after (right) irradiation, upper images; DIC images, lower images; fluorescence images).** PIT induced cell death with swelling, budding and rupture of the membrane as shown on the DIC images (middle and right). Fluorescence signals decreased after continuous NIR irradiation. Scale bar: 50 μm (original magnification, ×400).

Fluorescence microscopy was performed to confirm target-specific localization of Pan-IR700. Fluorescence was mainly localized to the cell membrane and lysosomes of the cells. During continuous NIR light exposure the cells demonstrated almost immediate swelling, budding and rupture of the membrane was observed leading to irreversible cell death (Figure 2).

### Effect of phototoxicity in response to Pan-IR700 mediated PIT

**Figure 3:**
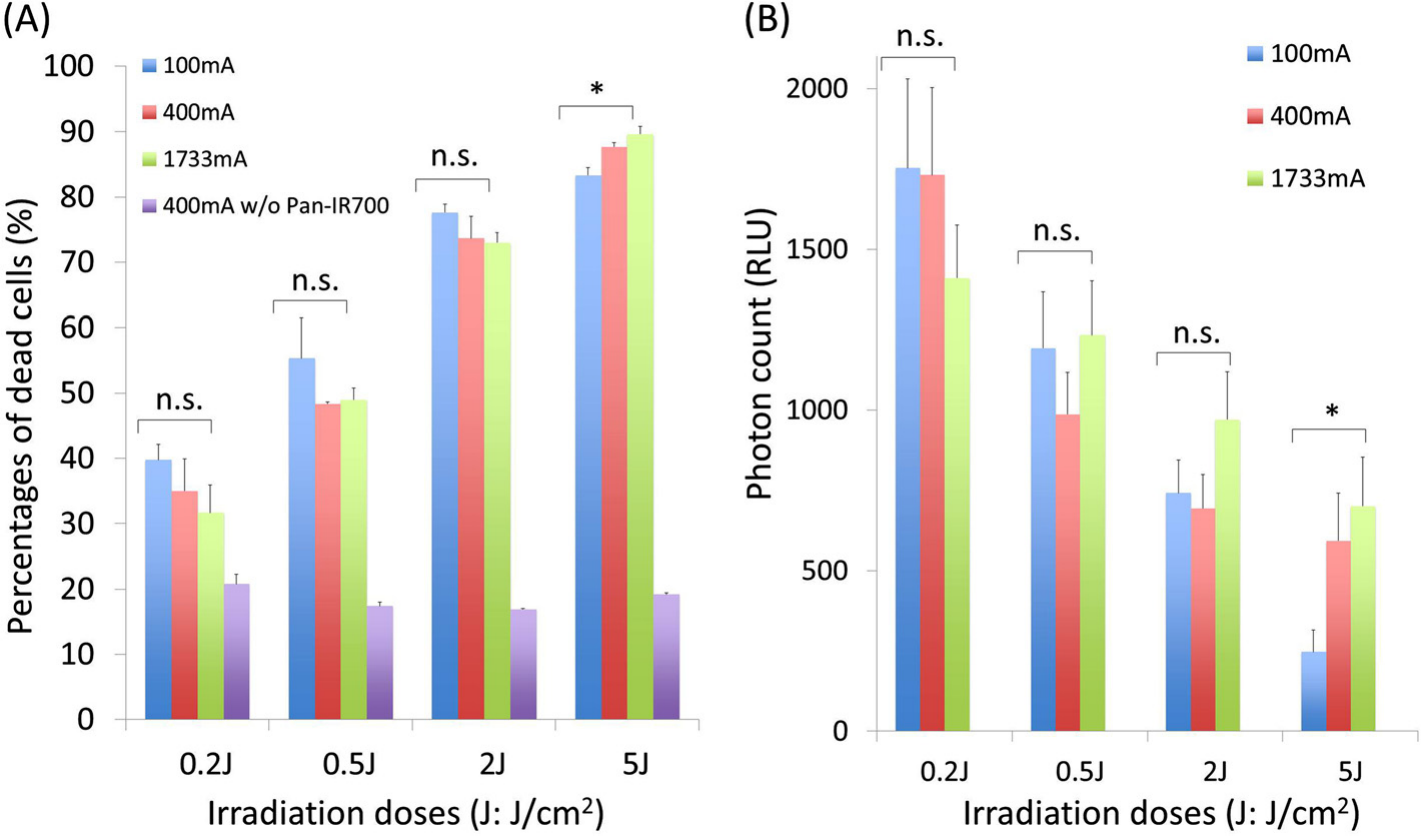
**Effect of phototoxicity in response to Pan-IR700 mediated PIT.** Cell viability was analyzed with **(A)** flow cytometory with dead staining using PI and **(B)** bioluminescence after LED light irradiation in MDA-MB-468luc cells. (n = 3, n.s. p > 0.05, *p < 0.05).

Flow cytometry showed that the ratio of dead cells increased from ~40 to ~85% as the light dose increased from 0.2 to 5 J/cm^2^ at every LED light power; 100, 400 and 1733 mA. At each light dose, no significant difference was observed in rate of cell death was seen among the 3 power levels (Figure 3A, 0.2 J/cm^2^: p > 0.05, 0.5 J/cm^2^: p > 0.05, 2 J/cm^2^: p > 0.05, 5 J/cm^2^: p = 0.014). Low levels of LED light irradiation dose (0.2 J/cm^2^) demonstrated relatively high bioluminescence signals. Bioluminescence signals decreased as exposure to NIR light dose increased. Bioluminescence signals were similar among the 3 different power levels at the same light dose (see Figure 3B, 0.2 J/cm^2^: p > 0.05, 0.5 J/cm^2^: p > 0.05, 2 J/cm^2^: p > 0.05, 5 J/cm^2^: p = 0.036). Larger doses of NIR light (5 J/cm^2^) resulted in correspondingly lower bioluminescence signals.

### Correlation between the concentration of Pan-IR700 and the irradiation dose

**Figure 4:**
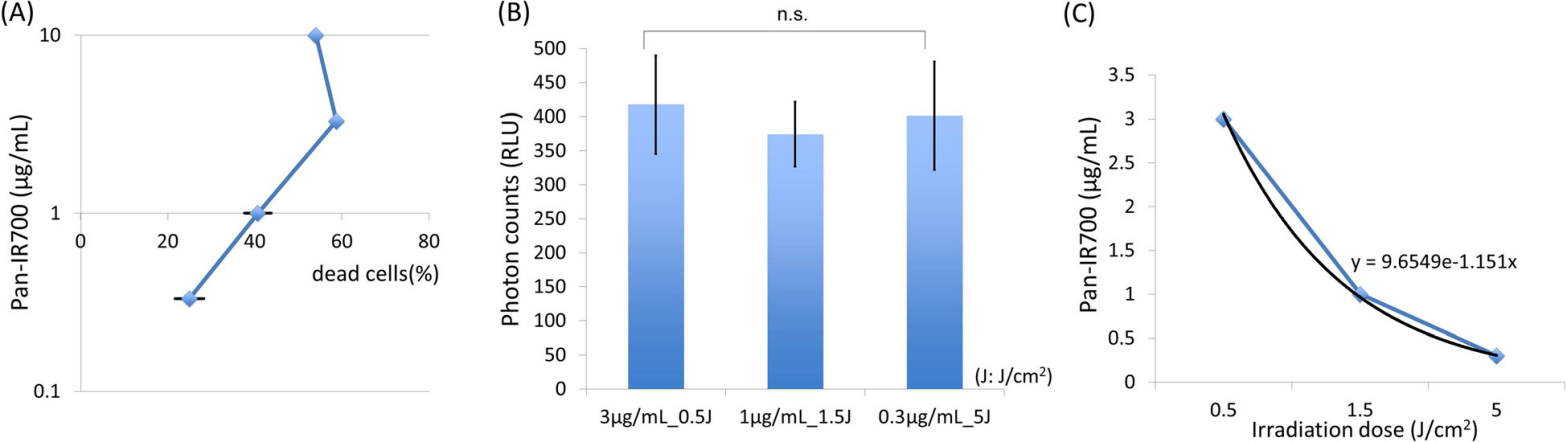
**Correlation between the concentration of Pan-IR700 and light dose for cell killing. (A)** At as constant light dose of 2 J/cm^2^, as the concentration of Pan-IR700 increased from 0.3 to 3 μg/mL, the percentage of dead cells increased. There was a positive linear correlation on a log scale between increasing dose and cell killing at a constant light dose (R^2^ = 0.94). Note, that above 3 μg/mL no further increases in cell death are seen due to saturation of the membrane antigens. **(B)** The same rate of cell killing (measured by bioluminescence imaging) could be achieved by any of the following combinations: (1) 3 μg/mL and 0.5 J/cm^2^, (2) 1 μg/mL and 1.5 J/cm^2^, and (3) 0.3 μg/mL and 5 J/cm^2^) resulting in no significant difference among these three groups (p > 0.05). **(C)** The correlation between light dose and the concentration of Pan-IR700 derived the following equation: [concentration of Pan-IR700] = 9.65e^-1.15 [irradiation dose]. The product of the light dose (in J/cm^2^) and the concentration of Pan-IR700 (in μg/mL) is a constant of 1.5.

A positive monoexponential correlation was seen between concentrations of Pan-IR700 ranging from 0.3 to 3 μg/mL and the cell death rate at a constant NIR light dose of 2 J/cm^2^ (Figure 4A, R^2^ = 0.94). Next we defined the effective dose parameter (1.5 J・μg/mL・cm^2^) by multiplying the Pan-IR700 concentration by the light dose. The cell death rate of the following combinations: (1) 3 μg/mL and 0.5 J/cm^2^, (2) 1 μg/mL and 1.5 J/cm^2^, and (3) 0.3 μg/mL and 5 J/cm^2^) showed no significant difference (Figure 4B, p > 0.05). The correlation between the light dose and the concentration of Pan-IR700 fit the following equation: [concentration of Pan-IR700 (μg/mL)] = 9.65e^-1.15 [irradiation dose (J/cm^2^)] (Figure 4C).

### *In vivo* photoimmunotherapy assessed by bioluminescence imaging

**Figure 5:**
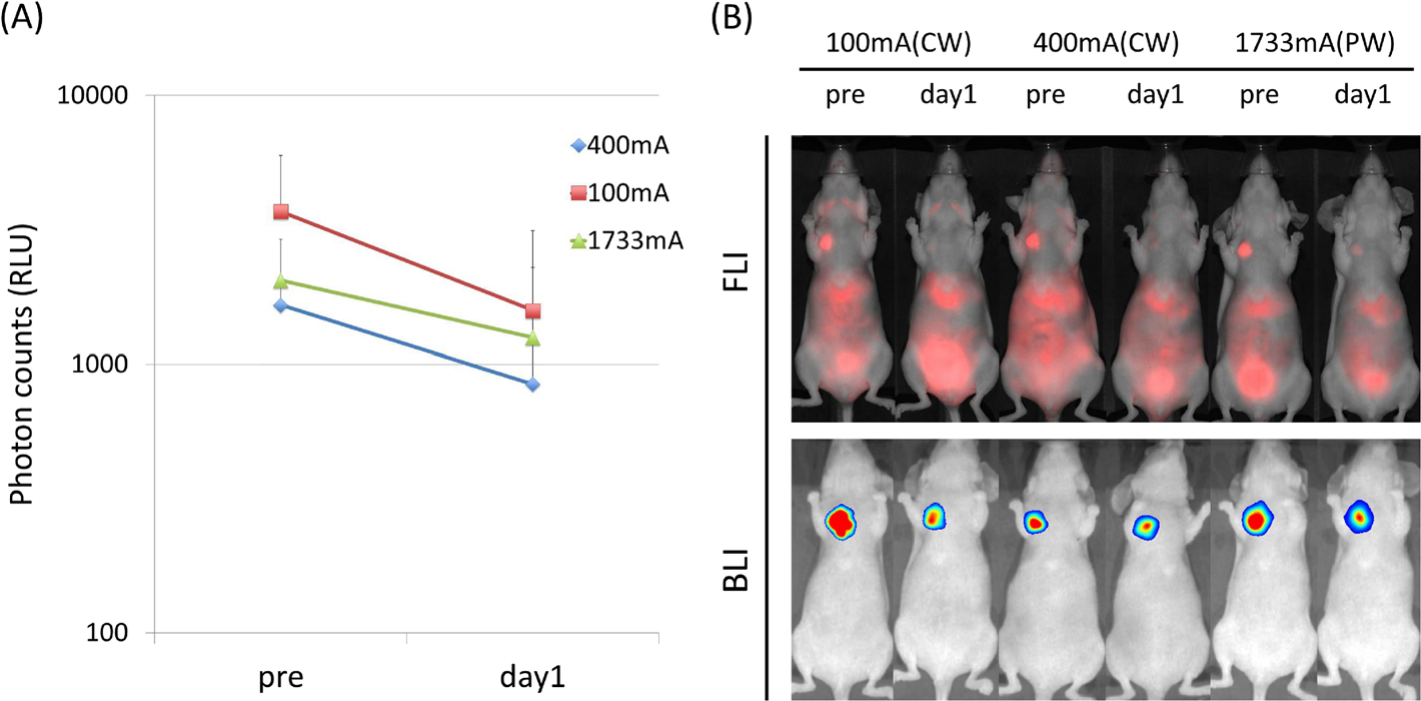
***In vivo *photoimmunotherapy in orthotopic breast tumors with a constant dose of 30 J/cm**^**2 **^**delivered by 100 mA (CW), 400 mA (CW) and 1733 mA (PW) LED light. (A)** Photon counts on bioluminescence images of tumors before and 1 day after LED irradiation. Photon counts of tumors in these three groups decreased around 60% (400 mA (CW), 60.1%; 100 mA (CW), 60.1%; 1733 mA (PW), 60.1%) 1 day after LED irradiation. **(B)***In vivo* fluorescence (upper row) and bioluminescence imaging (lower row) of orthotopic breast tumors before and 1 day after Pan-IR700 mediated PIT.

All NIR light exposure of 30 J/cm^2^ with three different light power levels (100 mA CW, 400 mA CW and 1733 mA PW) induced decreased bioluminescence signals of PIT-treated tumor compared with the pre-treated tumor. Bioluminescent photon counts of all these three groups decreased 61.4%; 100 mA (CW) 61% (400 mA (CW), 61.5%; 1733 mA (PW), 61.4%) 1 day after NIR light exposure (Figure 5).

## Discussion

These data demonstrate that increased cytotoxicity with PIT can be achieved by either increasing the dose of the mAb-IR700 (up to the saturating dose) or increasing the dose of light (up to the thermal limits). Moreover, a constant level of cell killing can be achieved by modulating the doses of both the conjugate and the light dose. These data also demonstrate that the power density (mW/cm^2^) of the light source is not as critical a factor as is the light dose (J/cm^2^) in determining the effectiveness of PIT. Moreover, the effect of a given light dose is independent of the manner in which the light is delivered, that is, either continuously or intermittently. Only at the relatively high light dose of 5 J/cm^2^, was a significant difference seen in dead staining and bioluminescence, among three power settings probably due to the long exposure times required to reach such a high light dose with low power NIR light (100 mA).

In addition to the effect of light dose, these data confirm that increasing concentrations of Pan-IR700 induced more cell death at a fixed light dose (2 J/cm^2^) (Figure 4A). However, above a concentration of 3 μg/mL of Pan-IR700 no further increases in cell death were seen suggesting that the EGFR antigens on the MDA-MB-468luc cell surface were saturated with Pan-IR700 at 3 μg/mL and therefore, higher doses did not result in further effectiveness (Figure 4).

MDA-MB-468 orthotopic breast tumors in mice administered Pan-IR700 showed ~60% decrease of bioluminescence signals after 30 J/cm^2^ of NIR light exposure. *In vivo* cytotoxicity data confirmed *in vitro* data that PIT-induced cytotoxicity is related to the product of the dose of the conjugate (up to the saturating dose) and the light dose.

In order to decrease either the dose of the conjugate or the light dose and achieve similar results, the spectral profile of the light source would need to be improved. In this study, we used an LED which emitted 690 ± 20 nm of NIR light. However, the absorbance spectrum of IR700 around 690 nm is sharper than the emission spectrum of this LED. Therefore, a large proportion of the exposed energy was not absorbed by IR700. Theoretically, we could improve the quantum efficiency of the light dose by using a laser light source tuned to emit a single 690 nm wavelength of light (± 5 nm).

Fluorescent proteins (FPs) are an excellent method for monitoring preclinical tumor growth *in vivo*[7-10]. FPs are stable and therefore ideal for longitudinal monitoring of photo-induced cancer therapies as has been shown previously [11,12]. However, FPs keep glowing before they are taken up and catabolized by macrophages *in vivo*[13] that takes hours after cell death [7]. In the current application the rapidity, with which PIT induces cell death, is difficult to detect with FPs as fluorescence would persist after cell death especially *in vitro*. Because firefly luciferin-based bioluminescence is an ATP-dependent process and ATP is released from damaged cells and hydrolyzed during or shortly after PIT, bioluminescence imaging is theoretically and practically an appropriate method for detecting *in vivo* PIT-induced cell necrosis [4]. Furthermore, FPs are excellent endogenous fluorescence emitters and/or singlet oxygen producers for operating as photo-dynamic therapy (PDT) agents. For selectively targeting cancer, recently reported technology is the use of telomerase promoter-regulated expression of various fluorescent proteins, which are induced with adenovirus-mediated gene transfection *in vivo*[11,12,14,15]. However, the requirement for virus-mediated *in vivo* gene transfection makes it unlikely to be translated for human use in the near term. In contrast, the PIT technology described here should be readily translatable as it requires the injection of an antibody-IR700 conjugate and exposure to non-thermal levels of NIR light.

## Conclusion

We demonstrate that cell killing with PIT is dependent on conjugate dose, up to the saturating dose, and the light dose. We found that a constant level of cell death could be achieved by varying the conjugate dose and light dose in such a way that the product was a constant. At a defined conjugate-light dose product, the cytotoxic effect of PIT is the same regardless of how the light is delivered (continuously or intermittently). This result suggests that the cytotoxic effect induced by PIT, which depends on the number of bound mAb-IR700 molecules and the exposed light dose, is highly predictable. Since the number of bound mAb-IR700 conjugates is roughly proportional to the intensity of IR700 fluorescence, this value could be used to calculate the appropriate effective and safe light dose in the future.

## Abbreviations

PIT: Photoimmunotherapy; NIR: Near infrared; mAb: Monoclonal antibody; FDA: Food and Drug Administration; CW: Continuous-wave; PW: Pulse-wave; LED: Light-emitting diode; ROI: Regions of interest.

## Competing interest

The authors declare that they have no competing interest.

## Authors’ contributions

TN conducted experiments, performed analysis and wrote the manuscript; KS, HH, RW, and TH, conducted experiments and performed analysis; PLC wrote the manuscript and supervised the project; and HK planned and initiated the project, designed and conducted experiments, wrote the manuscript, and supervised the entire project. All authors read and approved the final manuscript.

## Pre-publication history

The pre-publication history for this paper can be accessed here:

http://www.biomedcentral.com/1471-2407/14/389/prepub
